# The Toxicological Risk Assessment of Dermal Exposure of Patients Exposed to Lead and Cadmium Due to Application of Ointments with Marjoram Herb Extract (*Majoranae herbae extractum*)

**DOI:** 10.3390/ijerph20032701

**Published:** 2023-02-02

**Authors:** Kamil Jurowski, Mirosław Krośniak

**Affiliations:** 1 Laboratory of Innovative Toxicological Research and Analyses, Institute of Medical Studies, Medical College, Rzeszów University, Al. mjr. W. Kopisto 2a, 35-959 Rzeszow, Poland; 2 Department of Regulatory and Forensic Toxicology, Institute of Medical Expertises, Łódź, ul. Aleksandrowska 67/93, 91-205 Łódź, Poland; 3 Department of Food Chemistry and Nutrition, Medical College, Jagiellonian University, Medyczna 9, 30-688 Kraków, Poland

**Keywords:** heavy metals, toxicological risk assessment, marjoram herb, ICH Q3D (R2)

## Abstract

Potential heavy metal impurities (HMI) in pharmaceuticals/pharmaceutical products/drugs based on plant raw materials (e.g., herbs) are an important problem in the pharmaceutical industry; however, there is a lack of scientific articles on the comprehensive toxicological risk assessment of HMI in ointment applied dermally. To make the appropriate toxicological risk assessment, we consider: (1) the raw results of the levels of lead and cadmium in the ointments (metal per kg of mass), (2) one-time administration of the applied ointment, (3) daily exposure of Pb and Cd in the applied ointments according to the maximum daily dose of applied pharmaceuticals, including transcutaneous penetration, (4) human health risk assessment based on the USEPA model, and 5) the margin of exposure (MoE). The raw results indicated that lead (7.05–101.78 µg/kg) and cadmium (0.32–0.81 µg/kg) were present in all samples. The levels of analyzed HMI (independently of the producer and declared composition) were similar. Pb and Cd contamination associated with daily doses met the standards of the ICH Q3D (R2) guide on elemental impurities in pharmaceuticals, including the cutaneous route of administration. Taking into account the daily amount of lead and cadmium (ointment, ug/day) the results were satisfactory, confirming the safety of marjoram herb extract ointments available in Polish pharmacies according to the ICH guideline Q3D (R2) on elemental impurities. The HQ values obtained for Pb and Cd in all samples were less than 1; therefore, exposure to these HMI would not cause a health risk based on the USEPA model strategy. Furthermore, the obtained values of MoE for Pb and Cd in ointments were above 100, so exposure to these elements would not cause a health risk based on the MoE-based strategy. The original contribution of this work is that this is the first study on the triple approach strategy to evaluate the safety of heavy metal impurities in traditional herbal medicinal products applied dermally in solid form (ointments with marjoram herb extract). The research is novel and has not been previously published; The proposed procedure includes transcutaneous penetration of heavy metal (Pb and Cd) impurities described for the first time in the literature.

## 1. Introduction

*Origanum majorana*, L., herba (marjoram) is an active pharmaceutical ingredient (API) in traditional herbal medicinal products (THMPs) generally used among the European population for adjunctive rhinitis (runny nose). This THMP is applied as an ointment for the relief of irritated skin around the nostril [1]. Additionally, *Origanum majorana*, L., herba leaves have been used for the treatment of gastrointestinal disorders, coughs, and bronchial diseases [2,3]. Based on suggested dosages for a runny nose, a small amount of ointment is spread around the nostrils two to four times daily [1]. Hence, from a toxicological point of view, there is a potential health risk related to dermal exposure.

In this situation, appropriate toxicological risk assessment (TRA) based on recommendations from the European Medicines Agency (EMA) should be applied, such as the ICH Q3D (R2) guideline on elemental impurities in pharmaceuticals [4]. The appropriate TRA is essential because the monitoring of EI in final pharmaceuticals is a required step in new final pharmaceutical products [4]. However, these types of studies are rare but highly desirable for the pharmaceutical industry. Studies on HMI in pharmaceutical products including transcutaneous penetration of elements are very limited. This task is new and important because the ICH Q3D recommendation on elemental impurities (R1) for dermally applied drugs was published by the EMA on 25 September 2020 as a draft version (currently under public consultation) [4]. Surprisingly, only a limited number of studies has been conducted to assess systemic dermal absorption of lead and cadmium [4]. 

An interesting example of popular, cheap, and easily accessible pharmaceutical products applied dermally potentially presenting HMI is ointment with marjoram herb extract (*Majoranae herbae extractum*). The marjoram herb has been known and used in folk medicine for centuries [5]. Marjoram herb extract ointments are still used as a home remedy to adjunctively treat rhinitis, especially among the European population (e.g., Poland, Germany). An oil extract of marjoram is included in the Majorannae Unguentum pharmacopoeia ointment, used in the case of a runny nose to decrease swelling of the mucous membrane [6]. However, a potential problem with the application of marjoram herb extract in pharmaceutical products may be HMI (Pb, Cd). These metals are extremely hazardous because they are absorbed by the skin into the blood circulation. Impurities of lead and cadmium in agricultural soils are a potential problem. For example, at elevated blood concentrations, lead can affect cognitive development and reduce intellectual performance in children and can cause several cardiovascular dysfunctions in adults [7,8,9]. This toxic metal can also be a potential elemental impurity in the plant because it can be taken up by the roots of the plant [10,11]. The second most important heavy metal, cadmium, can cause kidney damage, affect skeletal and reproductive systems, and cause other health problems. Furthermore, because Cd is readily taken up by plants, an important issue is that the level of toxicity is in the range of 0.5–1 mg/kg of dry plant material, while crop plants can tolerate at least ten times this concentration in tissues [12]. Based on literature reviews [7,8,9,10,11,12], there is a possibility of this type of contamination in raw plant materials used for production of ointment with marjoram herb extract.

Therefore, the objective of this original article was the application of a triple strategy to assess the toxicological risks of lead and cadmium impurities in ointments with marjoram herb extract (*Majoranae herbae extractum*) used adjunctively in rhinitis that are available in Polish pharmacies. It should be emphasized that the originality of this work is that this is the first study to propose a comprehensive methodology for the toxicological evaluation of heavy metal impurities (Pb and Cd) in traditional herbal medicinal products in solid form (ointments).

The justifications for choosing these two metals were as follows:

- the fact that impurities of Pb and Cd in the final pharmaceuticals are the most important from a toxicological point of view (first class of elemental impurities in the ICH Q3D R1 guideline [4]);
- our analytical possibilities and scientific experience;
- the possibility of this kind of contamination in the raw plant materials used for the production of these THMPs based on a review of the literature [7,8,9,10,11,12];
- the rarity of this type of research in the field of comprehensive TRA of elemental impurities in final pharmaceutical products, such as ointments that include transcutaneous penetration of metals;
- the need to fill a gap in the scientific literature with respect to the control of these elemental impurities in pharmaceutical products applied dermally;
- popularization and application of integrated approaches for testing and assessment (IATA) to address current problems of regulatory toxicology to meet the needs of the pharmaceutical industry;
- the popularity of marjoram herb extract ointments as a remedy for runny nose in home practice among the European population.
- monitoring of heavy metal impurities in final traditional herbal medicinal products should be mandatory according to the ICH Q3D R1 elemental impurity guideline (ICH Q3D R1, 2019).

In this study, we analyzed marjoram herb extract ointments (*Majoranae herbae extractum*) available in Polish pharmacies (*n* = 5). It should be underlined that only five independent manufacturers produce this THMP in Poland. Therefore, this article is a case study. The results obtained represent a local picture only of the pharmaceutical market in Poland as an example of a country in the European Union. All the phytopharmaceuticals are registered in the EU as THMPs. The choice of this product (ointments with marjoram herb extract) was also dictated by the fact that these THMPs are very popular in Poland, based on: (1) the results of survey of the opinions of physicians and pharmacists (*n* = 94) from Kraków and Rzeszów; (2) interviews about popularity among patients (*n* = 105; 18 to 72 years old) from Niepoomice and Rzeszów (Poland), and (3) the literature, that includes pharmaceutical websites and blogs in Poland (accessed on June 2022).

Several modern analytical techniques are generally applied in element determination, including inductively coupled plasma mass spectrometry (ICP-MS), neutron activation analysis (NAA), and X-ray fluorescence-based technology (XRF); however, in our research, we applied the known and accepted atomic absorption spectroscopy (AAS) technique with Zeeman background corrections and electrothermal atomization. We used this analytical technique not only for our instrumentation, but also for its simplicity, availability, and well-integrated analytical background. The necessary step before the determination of Pb and Cd was a microwave-assisted digestion procedure. This was necessary to avoid the usual errors in determining the studied heavy metals in the presence of other elements and organic compounds (the effect of complex matrices). However, “dirty samples” with a matrix with high distilled water content, such as organic extracts from biological samples (like THMPs), increase the background and detection limits. The background correction device available for the atomic absorption spectrometer can overcome many of these problems. The background correction based on the Zeeman effect has proved to be very powerful; hence, we applied an atomic absorption spectrometer with Zeeman background corrections and electrothermal atomization. The novel contribution of this work is that this is the first study on the application of the triple approach strategy to evaluate the safety of heavy metal impurities in traditional herbal medicinal products applied dermally in solid form (i.e., ointments with marjoram herb extract). Our complex strategy has not been published previously. The proposed procedure includes transcutaneous penetration of heavy metal (Pb and Cd) impurities; therefore, it is very important that the pharmaceutical industry meets regulatory requirements (ICH Q3D recommendation on elemental impurities (R1) for dermally applied drugs).

## 2. Materials and Methods

### 2.1. Samples

In our study, we analyzed all available ointments in Poland (*n* = 5) with marjoram herb extract (*Majoranae herbae extractum*) sold as THMPs. This kind of herbal product is very popular in Polish pharmacies, especially for children under three years of age and for seniors. These types of products are monocomponent THMPs; therefore, other sources of EI are excluded. All products were collected from Kraków and Niepłomicie (Poland, Małopolska). The samples were collected in the period from March to May 2021. All the investigated samples were THMPs of individual manufacturers; it should be noted that only five manufacturers produce THMPs with marjoram herb extract in Poland. To maintain the highest methodological standards, each sample was coded (A, B, etc.). The description of all the samples analyzed is presented in the Supplementary Materials S1 (SM1).

### 2.2. Reagents

All reagents applied were of analytical grade. In the preparation of all solutions, demineralized water (Millipore) was used. Ultrapure demineralized water was obtained from the Milli-Q water purification system (Millipore, Bedford, MA, USA). Concentrated (65%) nitric acid from (Merck, SupraPur, Darmstadt, Germany) was applied. For our purposes, appropriate standard solutions were applied. Standard solutions of lead (Pb standard solution traceable to SRM from NIST–Pb (NO_3_)_2_ in 3.1% HNO_3_, 1000 mg/L CertiPUR^®^) and cadmium (Cd standard solution traceable to SRM from NIST–Cd (NO_3_)_2_ in 3.1% HNO_3_, 1000 mg/L CertiPUR^®^) were prepared by dilution of certified standard solutions (1000 μg/L; Merck, SupraPur, Darmstadt, Germany) of the corresponding metal ions.

The certified reference material (CRM; BCR-482; IRMM, Belgium) was a lichen-derived material. The second certified reference material (corn flour, INCT-CF-3) was purchased from the Department of Analytical Chemistry at the Institute of Nuclear Chemistry and Technology (Warsaw, Poland).

### 2.3. Toxicological Analysis Procedure

**Figure 1:**
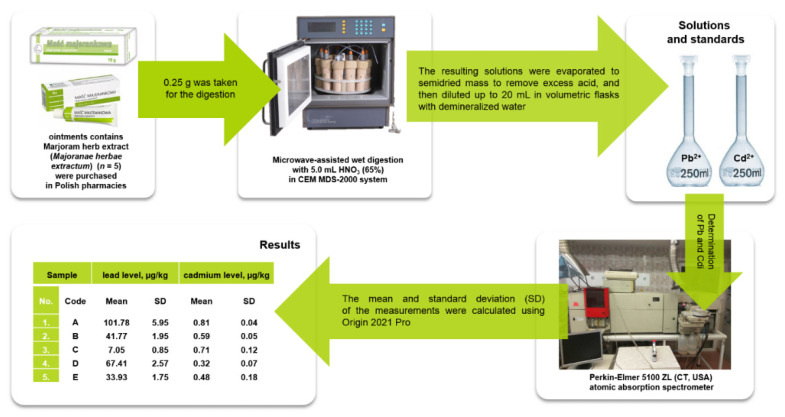
The toxicological analysis concept presented as a workflow diagram.

The toxicological analysis of the investigated elemental impurities was based on the applied studies described previously [13,14,15]. The toxicological analysis concept is presented schematically as a workflow diagram in Figure 1.

For the acid digestion of samples, microwave ovens MDS 2000 (CEM USA) and microwave-assisted digestion procedures were applied. All analyses were carried out using a Perkin–Elmer 5100 ZL atomic absorption spectrometer (CT, United States) with Zeeman background corrections and electrothermal atomization. The operating conditions of the applied analytical instrument are briefly described in Supplementary Materials S2 (SM2). Argon (purity 99.99%) was applied as a purge gas. The time temperature program of the analyzed graphite furnace is briefly described in Supplementary Materials S2 (SM2). The calibration curve was based on appropriate concentrations: 0.0; 1.0; 2.0; 5.0; 10.0 μg/L for Pb, and 0.0; 0.5; 1.0; 2.0 μg/L for Cd. The correlation coefficients (R) for Pb (0.988) and for Cd (0.988) obtained confirm that the analysis performed was accurate.

Recovery was 98.2% for Pb and 97.5% for Cd. The recovery levels were obtained as percentages of the determined level quotient and known amounts of the determined heavy metals. The calculated detection limits were 0.45 g/L for Pb and 0.15 g/L for Cd. The calculated limits of quantification (LOQs) were 0.96 g/L for Pb and 0.35 g/L for Cd. Additional information about the applied methodology is briefly summarized in Supplementary Materials S2 (SM2).

### 2.4. The Triple Approach Strategy for Comprehensive Safety Assessment of HMI

In this paper, we propose a three-stage strategy for a comprehensive assessment of heavy metal impurities, addressing the following important issues: (1) heavy metal impurities toxicological risk assessment, including the ICH Q3D (R2) elemental impurity guidance for transcutaneous absorption; (2) the U.S. Environmental Protection Agency (USEPA) model human health risk assessment, hazard index (HI); and (3) the external exposure (margin of external exposure, MoE) approach.

**Figure 2:**
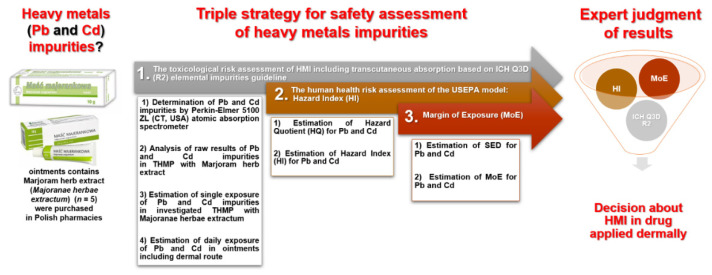
The applied three-stage strategy for safety assessment of HMI in ointments with marjoram herb extract (*Majoranae herbae extractum*) used adjunctively in rhinitis (runny nose) available in Polish pharmacies.

The idea of the applied three-stage strategy is presented schematically as a workflow diagram in Figure 2.

#### 2.4.1. HMI Toxicological Risk Assessment including Transcutaneous Absorption Based on the ICH Q3D (R2) Elemental Impurities Guideline

The cutaneous PDE should be applied in the assessment of toxicological risks. For this purpose, the ICH Q3D (R2) guideline involves a generic and conservative approach to the assessment of elemental impurities [4]. This approach is based on a systematic adjustment of parenteral PDE, which assumes 100% bioavailability, to derive an allowed daily exposure to the skin using a cutaneous modulating factor (in most cases intact/irritated skin 10; 100%/10% = 10) as in Equation (1) [4].

Cutaneous PDE = Parenteral PDE × CMF(1)

where:

Cutaneous PDE is the cutaneous permitted daily exposure;
Parenteral PDE is the parenteral permitted daily exposure;
and CMF is the cutaneous modifying factor.

#### 2.4.2. HMI Toxicological Risk Assessment, including Transcutaneous Absorption Based on the ICH Q3D (R2) Elemental Impurities Guideline

The second approach is the assessment of human health risk using the USEPA model applied to identify the exposure and assess risk to humans [13,14]. It should be noted that the human health risk models proposed by the US EPA have been successful and are adopted worldwide. The hazard quotient (HQi) represents the non-carcinogenic risk of a single contaminant according to Equation (2). It reflects the risk from all exposure pathways, i.e., inhalation of suspended particles through the mouth and nose, dermal absorption of trace elements in particles adhered to the exposed skin, and (though this does not apply to this situation) direct ingestion of particles.

HQi = DADi/RfDi(2)

where:

DADi—the dermal absorbed dose of HMI i (mg/kg bw/day);
RfDi—the reference dose of HMI i (mg/kg bw/day).

It should be noted that adverse health effects should be considered cautiously, and, if HQ > 1, this implies a high risk of toxicants with long-term health hazards; if HQ < 1, this indicates that there is no risk.

The numerator in Equation (2) is the dermal absorbed dose (DADi) for non-carcinogens, which can be calculated using the formula outlined by the US EPA represented by Equation (3).

DADi = (DAevent·EF·ED·EV·SA)/(BW·AT)(3)

where:

DADi—the dermal absorbed dose/dose absorbed through skin contact with element i (mg/kg bw/day);
DAevent—the dose absorbed per event (mg/cm^2^);
EF—the exposure frequency (application for no more than a week and 4 times/year; 28 days/year);
ED—the duration of exposure (30 years);
EV—the frequency of the event (4 times daily; 4 events/day);
SA—the available skin surface area or contact (average area of skin under the nose; 20 cm^2^);
BW—the average body weight (70 kg);
AT— the average time (30 years).

In Equation (3)., the value of DAevent (the amount absorbed per event) for each sample was obtained using Equation (4).

DAevent = Cointment·CF·AF·ABS(4)

where:

Cointment—the HMI concentration in ointment (mg/kg);
CF—the conversion factor (10–6 kg/mg) [14];
AF—the HMI adherence factor in ointment (0.07 mg/cm^2^) [14];
ABS—the absorption fraction (0.001) [14].

In Equation (2), the denominator is RfD, the estimated dose of daily exposure to humans that is likely not to have an appreciable risk of deleterious effects during a lifetime. Little information is available to calculate the risk assessment of Pb and Cd through dermal absorption. Therefore, the oral reference dose (RfDoral) was used as the fraction of the contaminant absorbed into the gastrointestinal tract to determine the reference dose for this study. It was the only and best assumption that could be made for the dermal absorption of the investigated HMI. These calculations were based on an analogous problem related to mercury [15,16]. The value of RfDdermal for each HMI was obtained using Equation (5).

RfD_dermal_ = RfD_oral_·ABSGI (5)

where:

RfD_dermal_—the dermal reference dose (mg/kg bw/day);
RfD_oral_—the oral reference dose (0.0035 mg/kg bw/day for Pb [17] and 0.001 mg/kg bw/day for Cd [18]);
ABSGI—the fraction of contaminant absorbed in the gastrointestinal tract (0.08 and 0.001 for Pb and Cd, respectively).

#### 2.4.3. The Margin of External Exposure (MoE) Approach

One of the important parameters in the safety evaluation of a dermally applied product (especially cosmetic ingredients) is the margin of exposure (MoE) (commonly known in the cosmetic industry as the margin of safety (MoS)), which is the relationship between a point of departure (PODsys; usually historical NOAEL or BMD values from oral or dermal studies) and an estimate of exposure [19], as represented in Equation (4).

MoE = POD_sys_/SED (6)

where:

POD_sys_—the point of departure (mg/kg bw/day);
SED—the systemic exposure dose (mg/kg bw/day).

SED was calculated according to the SCCS guidelines [19], using the percentage absorbed through the dermis—Equation (5).

SED = A (C/100%) (DA_p_100%) (7)

where:

SED—the systemic exposure dose (mg/kg bw/day);
A—the estimated daily exposure to a product per kg body weight, based on the amount applied and the frequency of application (mg/kg bw/day);
C—the concentration of the HMI under investigation in the finished product at the application site;
DA_p_—the dermal absorption expressed as a percentage of the test dose (assumed to be applied in real-life conditions).

For the most part, only repeated dose toxicity studies with oral exposure are available as surrogates for studies on dermal exposure. For comparison with PODsys, a systemic exposure dose (SED) for the dermal route is usually derived as the exposure estimate. It should be noted that the calculated MoE is compared with a reference MoE, which is comparable to the uncertainty/assessment factor used in risk and safety assessments to extrapolate from a group of test animals to an average human being, and subsequently from average humans to sensitive subpopulations. A default value of 100 (10 × 10), accounting for inter- and intraspecies differences, is generally accepted, and an MoE of at least 100, to indicate that an HMI is considered safe for use.

### 2.5. Statistical Approach

All the chemical analyzes were performed in triplicate. The mean and standard deviation (SD) as a percentage were calculated. The results of five independent replicates were expressed as mean ± standard deviation. Furthermore, descriptive statistics (minimum, maximum, mean, skewness, and kurtosis) using Origin 2021 Pro licensed by Jagiellonian University in Krakow were produced.

## 3. Results and Discussion

### 3.1. Lead and Cadmium Impurities in Ointments with Marjoram Herb Extract (Majoranae Herbae Extractum)

The triplicate results of each sample of marjoram herb extract (*Majoranae herbae extractum*) (*n* = 5) are shown in Table 1 as μg of HMI per kg of ointment. 

Lead levels were determined in all the investigated samples in the range 7.05–101.78 μg/kg. Lead levels were highest in sample A (101.78 ± 5.95 μg/kg)—approximately two/three times higher than in other samples (33.93–67.41 μg/kg), except sample C, where the level was lowest (7.05 ± 0.85 μg/kg). As mentioned in the Introduction, lead impurities can occur in agricultural soils; hence, they may be taken up by plant roots. Furthermore, because this heavy metal is readily absorbed by plants, an important issue is that the level of toxicity is in the range of 0.5–1 mg/kg of dry plant material, while crop plants tolerate at least ten times this concentration in tissue [12]. However, cadmium concentrations were extremely low in all ointments at similar levels (0.32–0.81 μg/kg).

### 3.2. Estimation of Exposure of Pb and Cd Impurities in Ointments with Marjoram Herb Extract Available in Polish Pharmacies

Based on the dosages (posology) recommended by the manufacturers for each THMP, a small amount of the product (ointment) should generally be placed around the nostrils. The approximate frequency of use is two to four times daily. The single dosage as the smallest amount (a drop of pea-size) should be applied around the nostrils with a clean finger. Based on the posology, it is not expected that the product would come into contact with the nasal mucosa. From this point of view, the average volume of one drop was calculated as approximately 0.25 g of each product. The calculated levels of lead and cadmium in the one-time administration of the applied ointments are presented in Table 2. These calculations are also necessary for the next level of risk assessment, the daily exposure of HMIs (the maximum daily dose of applied pharmaceuticals).

For appropriate TRA of this pharmaceutical product (i.e., ointment, transcutaneous absorption), calculations of the maximum daily quantity of pharmaceutical products used are obligatory. Usually, each THMP should be applied twice daily, especially after meals and before falling asleep. Daily external exposures to lead and cadmium through applied ointments were calculated considering maximum use during the day (Table 3).

External exposure to lead levels was variable (5.64–81.42 ng/day). However, external exposure to cadmium was relatively constant between ointments (0.256–0.648 ng/day). However, all the pharmaceutical products meet the standards of the ICH Q3D (R2) guideline on elemental impurities [4].

### 3.3. Estimated Daily Exposure to Lead in Ointments with Marjoram Herb Extract (Majoranae herbae extractum) via the Dermal Route

In typical toxicological risk assessment of pharmaceutical products applied orally [18,19,20], the calculated results of daily exposure should be compared with reference data, such as the allowed daily exposure (PDE) for HMI proposed by the ICH Q3D (R2) guideline on elemental impurities [4]. Because the products considered are applied dermally, the appropriate cutaneous PDE value for HMI should be calculated. Taking into account dermal contact and skin bioavailability (<1% [4]), the cutaneous PDE values for the elements investigated should be calculated using a cutaneous modification factor (CMF = 10 [4]). Therefore, the cutaneous PDE for HMI was calculated using Equation (1)—Table 4.

It should be underlined that HMI in dermal pharmaceuticals may be essential from a toxicological point of view due to the possible accumulation or absorption in the skin [21]. For example, lead is an example of a heavy metal that can pass from the skin to blood vessels and can then be transported to various critical organs [21,22]. Furthermore, it is well known that this element is poorly absorbed through intact skin (also with the presence of enhancers); for example, the absorption of Pb from lead oxide under occlusion in rats was less than 0.005% (measured by urinary Pb for twelve days after exposure) [23]. Our results (Table 2 vs. Table 3) indicated that all the products analyzed were characterized by results below the cutaneous PDE for this HMI (<50 µg/day). Therefore, all products met the requirements of the ICH Q3D (R1) elemental impurities guideline. On the other hand, all the results for cadmium (Table 3) were below the skin PDE value for this element (<20 µg/day, see Table 4.). However, it is strange and worrying that limited studies have been carried out on the dermal absorption of this heavy metal. Important studies by Wester et al. [24] were described with human cadaver skin in a diffusion cell model. In this article, information was provided on the penetration of 8.8 % (soil) and 12.7% (water) of the applied Cd dose into the skin (the plasma uptake of soil was 0.01% and 0.07% from water). A second study described by Lansdown et al. [25] showed that a solution of cadmium chloride (II) applied to the shaved skin of rats for 10 days triggered hyperkeratosis and acanthosis with occasional ulcerative changes [24]. In addition, the Cd content in the blood, liver, and kidneys increased. Godt et al. [26] provided an important summary of the dermal toxicity of this element, identifying two mechanisms to facilitate the absorption of this element in the skin. The first is based on the binding of available Cd^2+^ to cysteine sulfhydryl radicals in epidermal keratins. The second is induction and complexing with metallothionein. However, further relevant studies are needed on this topic.

### 3.4. Human Health Risk Assessment of the USEPA Model for Pb and Cd Impurities in Ointments with Marjoram Herb Extract (Majoranae herbae extractum) via the Dermal Route

Based on the USEPA equations [13,14] (Equations (1)–(5)), the exposure assessment results followed the order of: mouth intake > intake of direct contact with the skin > intake inhalation. However, in our situation, only dermal exposure should be considered. This is because the ointment is applied to the skin under the nose and possible contributions related to inhalation may be omitted because of their negligible nature. The assessment of human health risks related to Pb and Cd included consideration of noncarcinogenic health risks that were calculated using the hazard quotient (HQ). The RfDdermal value was not available in health risk databases, such as IRIS US EPA, ATSDR USA, and FDA, hence, calculations were made based on Equation (5). A summary of the human health risk assessment of the USEPA model is shown in Table 5. The HQ of Pb found in our study ranged from 5.64 × 10^−7^ to 8.14 × 10^−6^, and the HQ of Cd ranged from 1.08 × 10^−6^ to 7.17 × 10^−6^. The HQ values of all HMIs investigated in all samples were less than 1, therefore, exposure to Pb and Cd would not cause a health risk based on the USEPA model strategy.

### 3.5. The Margin of External Exposure (MoE) Approach

The last step of our proposed strategy was to estimate the external exposure margin for each HMI in all the samples investigated. First, the values of daily exposure to a product (A, mg/kg bw/day) were estimated based on the amount applied (0.25 g), the frequency of application (4/day), and the average weight of the adult person (70 kg). Then, based on the concentration of each HMI in the ointments and the maximal dermal absorption values (the worst-case scenario [19]), the SED values were calculated. The values of maximum dermal absorption were adopted from the appropriate literature. The percutaneous absorption of lead acetate after the use of hair-coloring preparations was almost nil, with a range between 0 and 0.3% of the dose applied to the skin [27]; therefore 0.3% was applied as the worst-case scenario. According to the ATSDR report for Cd [28,29], approximately 0.3–0.8% of cadmium is absorbed through the skin; hence, 0.8% was used for calculation. The last step was the calculation of the margin of exposure (MoE) based on the calculated values of SED and the appropriate PoD. The daily dietary intake of lead in adults corresponding to BMDL10 for blood pressure is 1.50 µg/kg bw/day, whereas the corresponding BMDL10 for renal effects (in adults, lead affects mainly blood pressure and kidney function, leading to chronic kidney disease) is 0.63 µg/kg bw/day [30]. Hence, for the calculation of Pb, a PoDsys value of 0.00063 mg/kg bw/day was applied. On the other hand, the EFSA Contaminant Panel calculated that the average daily dietary intake of cadmium should not exceed 0.36 µg/kg bw/day (TDI), which corresponds to BMDL_5_ = 0.004 mg/kg bw/day [31]. A summary of the results obtained is shown in Table 6.

It should be noted that, when the SED derived from dermal exposure is considered in the MoE calculation, the best approach should be the “external” NOAEL/BMDL derived from studies of dermal toxicity [32]. In most cases, PoDsys is usually derived from oral toxicity studies and oral absorption data should be considered to approximate the actual bioavailable internal dose. However, even after correcting for oral absorption, the MoE should generally be >100 [32]. Despite using conservative assumptions, the MoE values obtained for Pb and Cd in ointments with marjoram herb extract (*Majoranae herbae extractum*) were above 100; therefore, exposure to Pb and Cd would not cause a health risk based on the MoE-based strategy.

## 4. Conclusions

The levels of impurities of lead and cadmium in marjoram herb extract (*Majoranae herbae extractum*) used adjunctively in rhinitis (runny nose) available in Polish pharmacies (independently of the producer and the declared composition) are quite similar. It can be assumed that the lead and cadmium levels in all the ointments analyzed are at very low levels and, thus, are safe from the dermal route point of view. Their content in a single dose is also very low (ng/0.25 g) and is not a threat to patients (none of the ointments analyzed represents a health risk to patients). Furthermore, the results obtained for the investigated HMI meet the standards of the ICH Q3D (R2) guideline on elemental impurities, including transcutaneous penetration. Therefore, considering the daily dose of HMI analyzed (ointment, ng/day), the results are satisfactory and confirm the safety of ointments with marjoram herb extract (*Majoranae herbae extractum*) used adjunctively in rhinitis (runny nose). On the other hand, the HQ values of all the investigated HMIs, in all samples, were less than 1, therefore exposure to Pb and Cd would not cause a health risk according to the USEPA model strategy. Additionally, the MoE values obtained for Pb and Cd in ointments with marjoram herb extract (*Majoranae herbae extractum*) were greater than 100, so exposure to Pb and Cd would not cause a health risk according to the MoE-based strategy. 

It can be concluded that adjunctive ointments applied in rhinitis (runny nose) with *Majoranae herbae extractum* available in Polish pharmacies are safe for patients. However, these drugs should be monitored according to the presence of other EIs that have not yet been analyzed. The results obtained justify conducting this type of research because transcutaneous penetration is element-specific and chemical-species-specific, and each element would need to be experimentally evaluated under different conditions to develop an effective model. Furthermore, herbal medicines should be monitored for the presence of other important and potentially dangerous EI [29,30,31,32,33,34,35]. 

This is the first study of the application of a triple approach strategy in the safety assessment of heavy metal impurities in traditional herbs and medicinal products applied dermally in solid form (ointments with marjoram herb extract). However, other heavy metals (e.g., As and Hg) should also be investigated in the future for this kind of pharmaceutical product.

## Figures and Tables

**Table 1:** The levels of HMIs in the analyzed samples (ointments, μg/kg); SD—standard deviation.

Sample		Lead Level, µg/kg		Cadmium Level, µg/kg	
No.	Code	Mean	SD	Mean	SD
1.	A	101.78	5.95	0.81	0.04
2.	B	41.77	1.95	0.59	0.05
3.	C	7.05	0.85	0.71	0.12
4.	D	67.41	2.57	0.32	0.07
5.	E	33.93	1.75	0.48	0.18

**Table 2:** HMI levels in the analyzed samples (ointment, ng/0.25 g) one-time administration.

Sample		Lead Level, ng/0.25 g	Cadmium Level, ng/0.25 g
No.	Code	Mean	Mean
1.	A	20.36	0.16
2.	B	8.36	0.12
3.	C	1.41	0.14
4.	D	13.48	0.064
5.	E	6.79	0.096

**Table 3:** Daily external exposure of investigated HMIs in analyzed pharmaceuticals (ointment, ng/day).

Sample		Lead Level, ng/day	Cadmium Level, ng/day
No.	Code	Mean	Mean
1.	A	81.42	0.648
2.	B	33.42	0.472
3.	C	5.64	0.568
4.	D	53.93	0.256
5.	E	27.14	0.384

**Table 4:** Results of the calculation of the skin PDE for Pb and Cd.

HMI	PDE (µg/day) [4]	Cutaneous Bioavailability (%)	CMF	Cutaneous PDE (µg/day)
Pb	5.0	<1	10	50
Cd	2.0	<1	10	20

**Table 5:** Summary of the human health risk assessment of the USEPA model for Pb and Cd impurities in ointments with marjoram herb extract (Majoranae herbae extractum) via the dermal route.

**Pb Impurities**				
**Sample**	**RfD_dermal_**, **mg/kg bw/day**	**DA_event_**, **mg/cm^2^**	**DAD**, **mg/kg bw/day**	**HQ**
A	2.8 × 10^−4^	7.12 × 10^−11^	2.28 × 10^−9^	8.14 × 10^−6^
B	2.8 × 10^−4^	2.92 × 10^−11^	9.36 × 10^−11^	3.34 × 10^−6^
C	2.8 × 10^−4^	4.94 × 10^−12^	1.58 × 10^−10^	5.64 × 10^−7^
D	2.8 × 10^−4^	4.72 × 10^−11^	1.51 × 10^−9^	5.39 × 10^−6^
E	2.8 × 10^−4^	2.37 × 10^−11^	7.60 × 10^−10^	2.71 × 10^−6^
**Cd Impurities**				
**Sample**	**RfD_dermal_**, **mg/kg bw/day**	**DA_event_**, **mg/cm^2^**	**DAD**, **mg/kg bw/day**	**HQ**
A	10^−5^	5.67 × 10^−13^	1.81 × 10^−11^	1.81 × 10^−6^
B	10^−5^	4.13 × 10^−13^	1.32 × 10^−11^	1.32 × 10^−6^
C	10^−5^	4.97 × 10^−13^	1.59 × 10^−11^	1.59 × 10^−6^
D	10^−5^	2.24 × 10^−13^	7.17 × 10^−11^	7.17 × 10^−6^
E	10^−5^	3.36 × 10^−13^	1.08 × 10^−11^	1.08 × 10^−6^

**Table 6:** Summary of the safety assessment (SED and MoE) for impurities of Pb and Cd in ointments with marjoram herb extract (*Majoranae herbae extractum*) via the dermal route.

**Pb Impurities**						
**Sample**	**A**, **mg/kg bw/day**	**C**, **%**	**D_Ap_**, **%**	**SED**, **mg/kg bw/day**	**PoD_sys_**, **mg/kg bw/day**	**MoE**
A	14.28	1.02 × 10^−5^	0.3	4.362 × 10^−9^	0.00063	144,429
B	14.28	4.177 × 10^−6^	0.3	1.79014 × 10^−9^	0.00063	351,927
C	14.28	7.05 × 10^−7^	0.3	3.02143 × 10^−10^	0.00063	2,085,106
D	14.28	6.741 × 10^−6^	0.3	2.889 × 10^−9^	0.00063	218,069
E	14.28	3.39 × 10^−6^	0.3	1.45414 × 10^−9^	0.00063	433,245
**Cd impurities**						
**Sample**	**A**, **mg/kg bw/day**	**C**, **%**	**D_Ap_**, **%**	**SED**, **mg/kg bw/day**	**PoD_sys_**, **mg/kg bw/day**	**MoE**
A	14.28	8.1 × 10^−8^	0.8	9.26 × 10^−11^	0.004	43,209,920
B	14.28	5.9 × 10^−8^	0.8	6.74 × 10^−11^	0.004	59,322,093
C	14.28	7.1 × 10^−8^	0.8	8.11 × 10^−11^	0.004	49,295,824
D	14.28	3.2 × 10^−8^	0.8	3.66 × 10^−11^	0.004	109,375,109
E	14.28	4.8 × 10^−8^	0.8	5.49 × 10^−11^	0.004	72,916,740

## Data Availability

The datasets generated during and/or analysed during the current study are available from Kamil Jurowski (toksykologia@ur.edu.pl) upon reasonable request.

## Supplementary Materials

The following supporting information can be downloaded at: https://www.mdpi.com/article/10.3390/ijerph20032701/s1, Supplementary Materials S1 (SM1): the description of all the samples analyzed; Supplementary Materials S2 (SM2): additional information about the applied methodology.

## Abbreviations

AAS—atomic absorption spectrometry technique, API—active pharmaceutical ingredient, CBA—cutaneous bioavailability, CMF—cutaneous modification factor, EI—elemental impurities, ET AAS—electrothermal atomization atomic absorption spectrometry, HMI—heavy metal impurities; ICH Q3D—International Council for Harmonization of Technical Requirements for Pharmaceuticals for Human Use, MOE—margin of exposure, MHE—marjoram herb extract; PDE—permitted daily exposure, SD—standard deviation, US EPA—the United States Environmental Protection Agency, TRA—toxicological risk assessment, WHO—World Health Organization.
