# Supplementary material for: The Toxicological Risk Assessment of Dermal Exposure of Patients Exposed to Lead and Cadmium Due to Application of Ointments with Marjoram Herb Extract (Majoranae herbae extractum)
Source: Int J Environ Res Public Health. 2023 Feb 2;20(3):2701. doi: 10.3390/ijerph20032701 (PMC9916081; doi:10.3390/ijerph20032701)
Supplement: Supplementary file 1 [file ijerph-20-02701-s001.zip › Supplementary materials S1.pdf]

Supplementary Materials S1

Short description of analyzed samples.

Sample		latin description	license	note
No.	Code			
1.	A	*Majoranae herbae extractum*	IL-2800/LN	OTC
2.	B	*Majoranae ungentum*	20902	OTC
3.	C	*Majoranae ungentum*	IL-3036/LN	OTC
4.	D	*Majoranae herbae extractum/Majoranae ungentum*	IL-2601/LN	OTC
5.	E	*Majoranae ungentum*	IL – 3581/LN	OTC

OTC - over-the-counter.
