# Supplementary material for: The Toxicological Risk Assessment of Dermal Exposure of Patients Exposed to Lead and Cadmium Due to Application of Ointments with Marjoram Herb Extract (Majoranae herbae extractum)
Source: Int J Environ Res Public Health. 2023 Feb 2;20(3):2701. doi: 10.3390/ijerph20032701 (PMC9916081; doi:10.3390/ijerph20032701)
Supplement: Supplementary file 1 [file ijerph-20-02701-s001.zip › Supplementary materials S2.pdf]

Supplementary Materials S2

1. The AAS operating conditions.

Operating parameters	heavy metal	
	lead	cadmium
Wavelength [nm]	283.3	228.8
Slit width [nm]	0.7	0.7
Lamp current [mA]	8	5
Optimum working range [μg/kg]	1.0-10.0	0.02-0.20

2. Time-temperature program in the graphite furnace atomic absorption spectrometer in lead and cadmium determination.

Lead

Step	Temperature [°C]	Ramp [s]	Hold [s]	Gas Flow [mL/min]
1	120	1	30	250
2	950	10	20	250
3	1450	0	5	0
5	2400	1	2	250

Cadmium

Step	Temperature [°C]	Ramp [s]	Hold [s]	Gas Flow [$mL \cdot min^{-1}$]
1	120	10	25	250
2	300	5	15	250
3	1600	0	3	0
5	2400	1	2	250

3. A microwave-assisted digestion procedure

	Step 1	Step 2	Step 3	Step 4	Step 5
Power [W]	80	100	100	0	0
PSI	80	100	150	20	20
Time [minutes]	6:00	6:30	7:00	5:00	5:00
TAP [minutes]	2:00	5:00	5:00	0	0
Fan power [%]	100	100	100	100	100

Abbreviations: PSI – pounds-force per square inch; TAP – time at pressure min.
